# Impact of omega-3 fatty acids supplementation on lipid levels in pregnant women with previous pregnancy losses: a retrospective longitudinal study

**DOI:** 10.3389/fnut.2024.1439599

**Published:** 2024-08-29

**Authors:** Fangxiang Mu, Weijing Wang, Lin Liu, Ning Hu, Fang Wang

**Affiliations:** Department of Reproductive Medicine, Lanzhou University Second Hospital, Lanzhou, China

**Keywords:** dyslipidemia, pregnancy loss, pregnant women, omega-3 fatty acids, triglycerides

## Abstract

**Objective:**

This research aims to investigate the impact of omega-3 fatty acids supplementation on the lipid levels of pregnant women who have experienced pregnancy losses.

**Methods:**

This retrospective study analyzed data from pregnant women with previous pregnancy losses from two medical centers. Their lipid profiles were measured at least twice during pregnancy. According to the use of omega-3 soft gel capsules, participants were divided into the omega-3 group and the control group. We assessed the relationship between omega-3 fatty acids supplementation and longitudinal lipid levels during pregnancy using generalized estimating equations (GEE). Subsequently, we conducted subgroup analyses to delineate the profile of beneficiaries who received omega-3 fatty acids based on body mass index (BMI), age, menstrual regularity, number of previous pregnancy losses, number of previous live births, and educational level.

**Results:**

The omega-3 group included 105 participants, while the control group comprised 274 participants. Women in the omega-3 group started supplementation between 3.43 and 17.14 weeks of gestation. According to GEE analysis, supplementing omega-3 fatty acids significantly reduced triglyceride (TG) levels during pregnancy (adjusted β = −0.300, 95% CI -0.445 to −0.154, *p* < 0.001). No associations between omega-3 fatty acids supplementation and total cholesterol (TC), low-density lipoprotein cholesterol (LDL-C), or high-density lipoprotein cholesterol (HDL-C) levels were observed. Subgroup analyses revealed that omega-3 fatty acids supplementation was related to a reduction in TG levels among pregnant women with age of ≤35 years, a normal BMI (18.5–24.9 kg/m^2^), 1–2 previous pregnancy losses, no previous live births, or an educational level above high school.

**Conclusion:**

Supplementation with omega-3 fatty acids may significantly reduce TG levels, yet it does not seem to improve TC, LDL-C, or HDL-C levels in pregnant women with previous pregnancy losses.

## 1 Introduction

Pregnancy acts as a stress test that can predict a woman’s future cardiovascular health (1). Pregnancy loss, a prevalent pregnancy complication, affects approximately 23 million individuals annually (2). Studies have shown that a history of pregnancy losses is associated with an increased risk of cardiovascular disease later in life, with hyperlipidemia potentially serving as an intermediary (3). Specifically, women experiencing pregnancy losses may face inflammation and endothelial dysfunction, heightening their hyperlipidemia risk (4). The elevated risk of hyperlipidemia can, in turn, increase atherosclerosis and trigger cardiovascular events (5). Furthermore, cohort studies have demonstrated a correlation between gestational hyperlipidemia and adverse pregnancy outcomes, including preterm birth and preeclampsia. Those outcomes are identified as risk factors for future cardiovascular diseases (6). Therefore, managing lipid levels during pregnancy is essential to prevent adverse pregnancy outcomes and reduce the risk of subsequent cardiovascular disease. Currently, statins and similar first-line dyslipidemia drugs are usually avoided during pregnancy due to their teratogenic potential (6). However, omega-3 fatty acids, documented as safe for pregnant women, have shown promise in reducing maternal lipid levels (7, 8).

Mechanistically, omega-3 fatty acids can restrain the production and release of very-low-density lipoprotein (VLDL) particles, while also enhancing the clearance of triglyceride (TG) in both VLDL and chylomicrons by upregulating enzymes like lipoprotein lipase (LPL) (9). Consumption of omega-3 fatty acids may improve lipid profiles, insulin action, and inflammation-related gene expression by inhibiting pathways like protein kinase B and phosphoinositide 3-kinase, as well as modulating nuclear factor kappa-light-chain-enhancer function of activated B cells (NF-κB) pathway (10). Previous studies have evidenced that omega-3 fatty acids supplementation regulates lipid levels in pregnant women (11, 12). Helland and colleagues were the first to investigate the impact of marine omega-3 fatty acids supplementation versus omega-6 fatty acids on maternal and infant lipid levels in pregnant and lactating women. All 341 healthy pregnant women received 10 mL/day of omega-6 fatty acids (corn oil) or omega-3 fatty acids (cod liver oil) until 3 months postpartum. Results indicated a significant reduction in plasma lipid levels with omega-3 supplementation compared to omega-6 (12). Randomized controlled trials also demonstrated that omega-3 fatty acids can markedly lower TG levels and increase low-density lipoprotein cholesterol (LDL-C) and high-density lipoprotein cholesterol (HDL-C) levels, with good therapeutic tolerance for pregnant women (10, 13). Furthermore, hyperlipidemia during pregnancy may be more intense in gestational diabetes mellitus (GDM) women (14). Omega-3 fatty acids were shown to be effective in pregnant women with GDM (15, 16).

Similar to GDM women, those with a history of pregnancy losses may have a higher risk of hyperlipidemia, regardless of early or late losses, spontaneous abortion or not (4). A history of multiple pregnancy losses significantly increases the risk of hypertriglyceridemia, low HDL-C levels, and hyperglycemia (17). Animal experiments have shown that the lipid metabolic synthesis pathways are altered in the serum of incomplete medical abortion rats (18). Additionally, the pooled risk of pregnancy losses can be as high as 15.3%, and a history of pregnancy losses may be a characteristic of some pregnant women (2). However, this high-risk population has not received much attention in the literature. Therefore, we aim to explore the relationship between omega-3 fatty acids supplementation and the lipid levels (total cholesterol (TC), TG, LDL-C, HDL-C) of women with previous pregnancy losses.

## 2 Methods

### 2.1 Participants and study design

We retrospectively analyzed data from patients who visited the Reproductive Center of Lanzhou University Second Hospital between September 2019 and February 2022, and those who visited the Center for Reproductive Medicine at the First Affiliated Hospital of Chongqing Medical University between October 2022 and December 2023. Participants were assigned to the omega-3 group if they used omega-3 fatty acids capsules during pregnancy. Those capsules were produced by the Chengdu Suncadia Medicine Co., Ltd. and approved by China National Medical Products Administration (approval number H20223269). Each capsule contains 465 mg of eicosapentaenoic acid (EPA) ethyl ester and 375 mg of docosahexaenoic acid (DHA) ethyl ester. The baseline data were collected before conception, representing pregestational levels. All patients had their TC, TG, LDL-C, and HDL-C measured at least twice during the medication period. The timing of lipid measurements of the omega-3 and control groups was concentrated in the first and second trimesters.

### 2.2 Inclusion and exclusion criteria

To participate herein, women must meet the following eligibility criteria: (1) have a history of pregnancy loss; (2) have undergone at least two blood lipid assessments during pregnancy; and (3) have provided informed consent.

Women will be excluded under any of the following conditions: (1) history of acute or chronic pancreatitis, symptomatic gallstone disease (excluding those treated via cholecystectomy); (2) recent (within the past 6 months) cardiovascular events including acute coronary syndrome, angina pectoris, transient cerebral ischemia, or unstable congestive heart failure requiring treatment, vascular reconstructive surgery, aortic aneurysm, nephrotic syndrome, or diseases affecting the lungs, liver, or biliary tract; (3) GDM; (4) poorly controlled hypertension, defined as three consecutive clinic visits where systolic blood pressure is consistently ≥140 mmHg and/or diastolic blood pressure is consistently ≥90 mmHg while at rest; (5) a history of gastrointestinal surgery or dysfunction; (6) substance abuse or alcoholism within the previous year; or (7) use of statins, beta-blockers, niacin, cholesterol absorption inhibitors, and Chinese herbal medicines known to affect lipid levels during the study period.

### 2.3 Biochemical assays

Laboratory analyses were conducted on the serum or serum from 12-h fasting blood samples. TC and TG were determined by an enzymatic colorimetric assay (Roche Diagnostics). LDL-C and HDL-C were quantified using the homogeneous enzymatic colorimetric assay (Roche Diagnostics).

### 2.4 Statistical analysis

We used the SPSS Statistics 25.0 (SPSS Inc., Chicago, IL, United States) for statistical analysis. We reported continuous variables as means ± standard deviation (SD), and categorical variables as frequencies (percentages). To compare baseline characteristics among participants, independent samples t-tests or Mann–Whitney U tests were used for continuous variables, while Fisher’s exact tests or chi-square tests were used for categorical variables.

To evaluate the impact of omega-3 fatty acids supplementation on lipid levels during pregnancy, we employed generalized estimating equations (GEE), a method suitable for analyzing repeated measures, and adjusted for age, body mass index (BMI), number of previous pregnancy losses, and menstrual regularity. Subgroup analyses were also conducted using the GEE approach, with the same covariates included. GEE is a robust estimation technique that addresses the potential violation of the sample independence assumption often encountered using traditional univariate linear regression models (19). GEE boasts a key advantage: it does not necessitate accurate distribution specification but only of the mean structure. Additionally, GEE allows for variance structure, a critical factor in managing the correlation of longitudinal data analysis. We proceeded to create effect plots of the generalized estimating model using R studio version 2023.03.1 + 446 and R version 4.3.3 (R Foundation for Statistical Computing, Vienna, Austria). All tests were conducted using a two-sided approach, and a *p*-value below 0.05 was considered statistically significant.

## 3 Results

### 3.1 Demographic characteristics

A total of 379 participants were enrolled in this study. Of these, 312 patients (out of 331) were recruited from the Lanzhou University Second Hospital and 67 (out of 75) from the First Affiliated Hospital of Chongqing Medical University. The average daily intake of capsules was 3.18 ± 0.17 at the former and 1.97 ± 0.02 at the latter. Supplementary Table S1 presents the baseline characteristics of participants from both hospitals, with significant differences in menstrual regularity and previous pregnancy loss.

The 379 participants were divided into the omega-3 group (*n* = 105) and the control group (*n* = 274). Women in the omega-3 group started supplementation in the first and second trimesters, beginning between 3.43 and 17.14 weeks of gestation. Both groups had comparable baseline characteristics, including age, BMI, age at menarche, number of previous live births, education level, TG, HDL-C, or LDL-C levels. However, the omega-3 group had a significantly higher rate of irregular menses and TC level. For the number of pregnancy losses, the omega-3 group had a higher rate of once (31.4%) than the control group (9.5%), but a lower rate for twice and three or more times (twice: 53.3% vs. 69.7%; three or more times: 15.2% vs. 20.8%). In addition, the mean gestational age at lipid measurements for the omega-3 group was significantly earlier than the control group, at 12.67 ± 7.64 versus 17.51 ± 9.16. This difference was accounted for in the GEE analysis. Detailed information can be found in Table 1.

**Table 1:** Baseline characteristics of participants.

Characteristic	Omega-3 (*n* = 105)	Control (*n* = 274)	*p*-value
Age, years	31.19 ± 3.77	30.71 ± 3.49	0.243
Age at menarche	13.41 ± 1.21	13.34 ± 1.13	0.596
*Body mass index, kg/m^2^			0.409
< 18.5	6 (5.7)	25 (9.1)	
18.5–24.9	83 (79.0)	200 (73.0)	
≥ 25	16 (15.2)	49 (17.9)	
Menstrual regularity			0.004
Yes	81 (77.1)	243 (88.7)	
No	24 (22.9)	31 (11.3)	
Number of previous pregnancy losses			< 0.001
1	33 (31.4)	26 (9.5)	
2	56 (53.3)	191 (69.7)	
≥ 3	16 (15.2)	57 (20.8)	
Number of previous live births			0.286
0	94 (89.5)	232 (84.7)	
1	9 (8.6)	39 (14.2)	
2	2 (1.9)	3 (1.1)	
Education level			0.250
Below high school	9 (8.6)	39 (14.2)	
Senior high school	14 (13.3)	43 (15.7)	
Above high school	82 (78.1)	192 (70.1)	
*TC, mmol/L	4.42 ± 0.81	3.91 ± 0.67	< 0.001
*TG, mmol/L	1.14 ± 0.54	1.16 ± 0.67	0.912
*LDL-C, mmol/L	2.52 ± 0.89	2.47 ± 0.61	0.805
*HDL-C, mmol/L	1.65 ± 0.58	1.42 ± 0.29	0.065
Gestational weeks of lipid measurements	12.67 ± 7.64	17.51 ± 9.16	< 0.001

Variables are expressed as mean ± SD or n (%). TC, total cholesterol; TG, triglyceride; LDL-C, low-density lipoprotein cholesterol; HDL-C, high-density lipoprotein cholesterol.

* Represents pre-gestational levels.

### 3.2 Impact of omega-3 fatty acids on lipid levels

**Figure 1:**
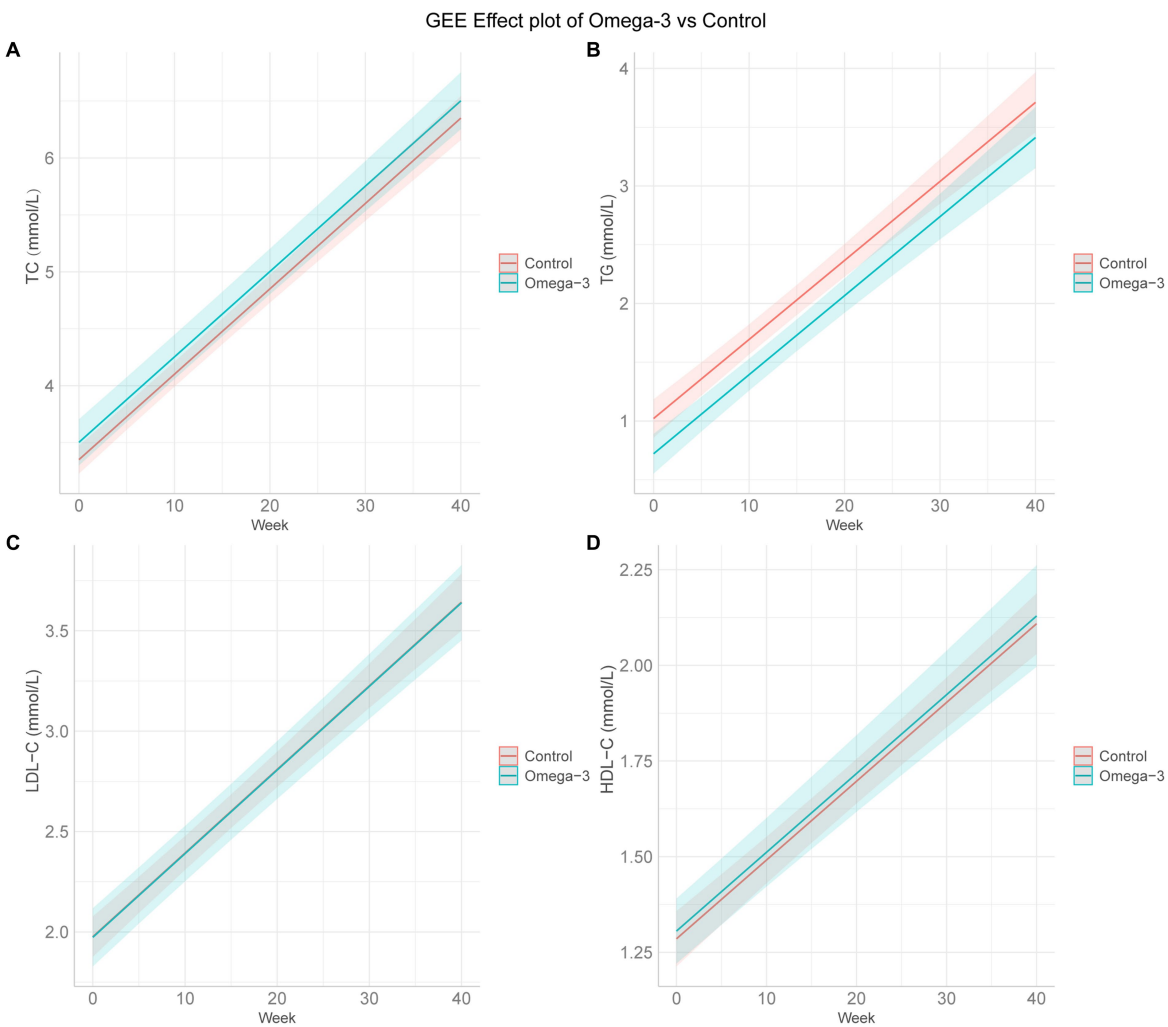
GEE effect plots of omega-3 fatty acids and control group. **(A–D)** The GEE effect plots of omega-3 fatty acids and control group on TC, TG, LDL-C, and HDL-C in order. GEE, generalized estimating equation; TC, total cholesterol; TG, triglyceride; LDL-C, low-density lipoprotein cholesterol; HDL-C, high-density lipoprotein cholesterol. The GEE model adjusted for age, body mass index, the number of previous pregnancy losses, and menstrual regularity.

Based on GEE analysis results, omega-3 fatty acids supplementation significantly decreased TG levels during pregnancy, both before and after adjusting for age, BMI, and history of pregnancy loss (unadjusted β = −0.288, 95% CI −0.436 to −0.140, *p* < 0.001; adjusted β = −0.300, 95% CI −0.445 to −0.154, *p* < 0.001). However, no meaningful impacts were on TC, LDL-C, or HDL-C levels (Table 2; Figure 1).

**Table 2:** Effects of omega-3 fatty acids on TC, TG, LDL-C, HDL-C levels.

	GEE model					
	Unadjusted intervention effect			Adjusted intervention effect^a^		
	β (95% CI)	*P*-value	QIC	β (95% CI)	*P*-value	QIC
TC	0.159 (−0.028, 0.346)	0.095	1477.228	0.152 (−0.025, 0.329)	0.092	1477.768
TG	−0.288 (−0.436, −0.140)	<0.001	2449.193	−0.300 (−0.445, −0.154)	< 0.001	2433.787
LDL-C	0.004 (−0.128, 0.135)	0.957	918.334	−0.003 (−0.127, 0.120)	0.960	927.898
HDL-C	0.018 (−0.084, 0.119)	0.729	320.586	0.020 (−0.084, 0.124)	0.703	369.630

Variables are given as beta (β) and 95% confidence intervals (CIs). GEE, generalized estimating equations; TC, total cholesterol; TG, triglyceride; LDL-C, low-density lipoprotein cholesterol; HDL-C, high-density lipoprotein cholesterol; QIC, Quasi-likelihood under the independence model criterion.

a The GEE model adjusted for age, body mass index, the number of previous pregnancy losses, and menstrual regularity.

### 3.3 Subgroup analyses of omega-3 fatty acids and lipid levels

We performed subgroup analyses according to maternal age, BMI, menstrual cycle regularity, number of previous pregnancy losses, number of previous live births, and education level. The results showed that omega-3 fatty acids consumption significantly lowered TG levels among pregnant women who were under 35 years of age (β = −0.319, 95% CI −0.467 to −0.171, *p* < 0.001), had a BMI between 18.5–24.9 kg/m^2^ (β = −0.288, 95% CI −0.463 to −0.114, *p* = 0.001), regular menstrual cycles (β = −0.293, 95% CI −0.452 to −0.135, *p* < 0.001), no previous live births (β = −0.324, 95% CI −0.480 to −0.169, *p* < 0.001), and experienced pregnancy losses less than three times (one loss: β = −0.534, 95% CI −0.932 to −0.137, *p* = 0.008; two losses: β = −0.360, 95% CI −0.530 to −0.191, *p* < 0.001). Participants with education beyond high school also benefit from omega-3 fatty acids for lowering TG levels (β = −0.308, 95% CI −0.475 to −0.140, *p* < 0.001) (Supplementary Table S2).

TC levels showed significant increases in groups older than 35 years (β = 0.585, 95% CI 0.082–1.087, *p* = 0.023), three or more previous pregnancy losses (β = 0.483, 95% CI 0.191–0.775, *p* = 0.001), and education degree above high school (β = 0.258, 95% CI 0.082–0.435, *p* = 0.004) (Supplementary Table S3).

Moreover, Supplementary Table S4 shows omega-3 fatty acids can notably increase LDL-C levels in participants with two previous live births (β = 0.292, 95% CI 0.092–0.492, *p* = 0.004). Then, subgroup analysis further evidenced that omega-3 fatty acids supplementation had no association with HDL-C levels during pregnancy, as shown in Supplementary Table S5.

## 4 Discussion

Given that dyslipidemia is linked to various adverse pregnancy outcomes and women with previous pregnancy losses have an elevated risk of hyperlipidemia compared to the general pregnant population, it is crucial to give more attention to those women to prevent and treat potential adverse effects caused by lipid abnormalities timely (4, 20, 21). In this longitudinal study involving Chinese women with a history of pregnancy losses, we comprehensively explored the effectiveness of omega-3 fatty acids in regulating lipid levels during pregnancy. The results indicated that omega-3 fatty acids notably decreased TG levels and did not affect TC, LDL-C, or HDL-C levels. Subgroup analysis further suggested that the decrease in TG levels was more pronounced in women with an age of ≤35 years, a normal BMI, 1–2 previous pregnancy losses, and a higher education level beyond high school. Our research may have implications for lipid regulation during pregnancy and the healthy pregnancy of women who unfortunately experienced pregnancy losses.

During the third trimester of pregnancy, a woman’s plasma TG levels can increase by 2–4 times. Certain individuals may experience abnormally high TG levels due to increased production of TG-rich lipoproteins, mainly VLDL, and decreased activity of enzymes such as LPL and hepatic lipase (22, 23). These changes can lead to severe hypertriglyceridemia and even trigger pancreatitis in women with disrupted lipoprotein metabolism (24). Although the mechanisms causing hypertriglyceridemia are not fully understood, certain genetic mutations, such as Apolipoprotein E mutations, APOA5 mutation, and APOC2 mutation, may be involved, leading to protein defects and function loss (24). Clinically, omega-3 fatty acids have been reported to lower serum TG levels by 25–30% (25). Supplementation with fish oil in overweight and obese mothers increased the omega-3 index of maternal erythrocytes and decreased plasma TG levels in both mothers during pregnancy and infants at 3 months of age (11). Our study observed a negative correlation between omega-3 fatty acids and TG. It is believed that omega-3 fatty acids can lower TG concentrations by reducing its synthesis and secretion and enhancing the clearance of TG from VLDL particles (9). Although the special mechanisms by which omega-3 fatty acids affect lipid metabolism remain unclear, several possible mechanisms for their impact on TG levels have been proposed: (1) omega-3 fatty acids may promote the β-oxidation of fatty acids, thereby decreasing the available substrates necessary for TG and VLDL synthesis (9); (2) omega-3 fatty acids could lower TG levels by limiting the transport of non-esterified fatty acids (NEFA) to the liver and boosting phospholipid production there (26); (3) research suggests that they may reduce hepatic fat production by inhibiting the expression of sterol regulatory element-binding protein-1c, reducing enzyme activity related to cholesterol, fatty acids, and TG synthesis (27, 28); (4) they inhibit key enzymes associated with hepatic TG synthesis, like phosphatidate phosphatase and diacylglycerol acyltransferase (29); and (5) finally, they have been shown to increase LPL activity to accelerate chylomicron TG clearance, and EPA and DHA are equally effective (30).

Our research showed that omega-3 fatty acids supplementation was unrelated to TC, LDL-C, and HDL-C in women with a history of pregnancy loss. There is limited data on omega-3 fatty acids intake in this special pregnant women, and evidence from other pregnant groups is contradictory (16, 31, 32). Faraji et al. investigated the impact of fish oil supplementation during pregnancy on the lipid profile. Ninety two healthy pregnant women were randomly assigned to the supplementation group (*n* = 45, fish oil capsules, 1,000 mg/day) and the placebo group (*n* = 47). From the 21st week of pregnancy, one fish oil capsule or placebo was taken daily until delivery. The results showed that fish oil supplementation was not associated with the plasma lipid profile. Faraji believed that the effect of omega-3 fatty acids nutritional intervention may be neutralized by physiological factors that maintain high levels of circulating TG (33). Conversely, Jamilian et al. found that supplementing with fish oil for 6 weeks significantly reduced TG levels, and increased LDL-C and HDL-C levels, but did not impact TC levels during pregnancy. A potential mechanism is that omega-3 fatty acids supplementation may upregulate the expression of PPAR-γ and downregulate the expression of LDL receptors in peripheral blood mononuclear cells (10). Variations in findings across studies may stem from differences in treatment duration, timing to start, and dosage of omega-3 fatty acids supplements. Other factors include whether the studies involved healthy or ill pregnant women, the mothers’ prior status of polyunsaturated fatty acids, and the presence of pregnancy complications. The effects of omega-3 fatty acids on pregnant women with previous pregnancy losses must be validated by more rigorous prospective studies or randomized controlled trials.

Our study has advantages. It provides new evidence for the use of omega-3 fatty acids during pregnancy to prevent hyperlipidemia and extends the population to women with a history of pregnancy losses, a high-risk group for lipid abnormalities (4). Then, unlike previous studies that measured lipid levels at specific pregnancy stages, our longitudinal study focused on lipid changes throughout pregnancy, potentially evaluating omega-3 fatty acids effects more comprehensively. However, our study has some limitations. Although our study demonstrated that omega-3 fatty acids supplementation lowered lipid levels in pregnant women with a history of pregnancy losses, we acknowledge limitations in our intervention data such as lacking data on supplementation duration. Then, we may underestimate the dietary influences on our findings. Participants’ natural omega-3 fatty acids intake through their diets and adherence to specific dietary patterns, such as the Mediterranean diet, were not comprehensively assessed, which may introduce confounding bias. Future studies should include stringent dietary assessments to distinguish the effects of omega-3 fatty acids supplementation from other dietary influences. Furthermore, the mean gestational age of lipid measurements in the omega-3 group was earlier than in the control group, potentially skewing the results. However, the GEE model accounted for gestational week, ensuring that lipids were compared at the same gestational age, mitigating the concern about differences in gestational age.

## 5 Conclusion

Our study suggests that omega-3 fatty acids may reduce TG levels in pregnant women with previous pregnancy losses, but they may not affect levels of TC, LDL-C, and HDL-C.

## Data Availability

The raw data supporting the conclusions of this article will be made available by the authors, without undue reservation.
