# Supplementary material for: Impact of omega-3 fatty acids supplementation on lipid levels in pregnant women with previous pregnancy losses: a retrospective longitudinal study
Source: Front Nutr. 2024 Aug 29;11:1439599. doi: 10.3389/fnut.2024.1439599 (PMC11390446; doi:10.3389/fnut.2024.1439599)
Supplement: Supplementary file 1 [file Table_1.DOCX]

Supplementary Material

# Supplementary Tables

**Table S1.** Baseline characteristics of participants at two hospitals.

| Characteristic | Hospital of Chongqing Medical University  (n = 67) | Lanzhou University Second Hospital  (n = 312) | P-value |
| --- | --- | --- | --- |
| Age, years | 31.55 ± 4.07 | 30.69 ± 3.44 | 0.074 |
| Body mass index, kg/m^2^ | 22.15 ± 2.51 | 22.37 ± 3.06 | 0.528 |
| Age at menarche | 13.52 ± 1.19 | 13.32 ± 1.14 | 0.200 |
| Menstrual regularity |  |  | <0.001 |
| Yes | 47 (70.1) | 277 (88.8) |  |
| No | 20 (29.9) | 35 (11.2) |  |
| Number of previous pregnancy losses |  |  | <0.001 |
| 1 | 22 (32.8) | 37 (11.9) |  |
| 2 | 33 (49.3) | 214 (68.6) |  |
| ≥ 3 | 12 (17.9) | 61 (19.6) |  |
| Number of previous live birth |  |  | 0.194 |
| 0 | 59 (88.1) | 267 (85.6) |  |
| 1 | 6 (9.0) | 42 (13.5) |  |
| 2 | 2 (3.0) | 3 (1.0) |  |
| Education level |  |  | 0.521 |
| Below high school | 6 (9.0) | 42 (13.5) |  |
| Senior high school | 9 (13.4) | 48 (15.4) |  |
| Above high school | 52 (77.6) | 222 (71.2) |  |

**Table S2**. Subgroup analyses of omega-3 fatty acids and triglycerides.

|  | **β (95% CI)** | **P‑value** |
| --- | --- | --- |
| **Age, years** |  |  |
| ≤ 35 | -0.319 (-0.467, -0.171) | < 0.001 |
| > 35 | 0.086 (-0.175, 0.348) | 0.517 |
| **Body mass index, kg/m^2^** |  |  |
| < 18.5 | -0.205 (-0.424, 0.014) | 0.067 |
| 18.5-24.9 | -0.288 (-0.463, -0.114) | 0.001 |
| ≥ 25 | -0.374 (-0.764, 0.015) | 0.059 |
| **Menstrual regularity** |  |  |
| Yes | -0.293 (-0.452, -0.135) | < 0.001 |
| No | -0.419 (-0.819, -0.019) | 0.040 |
| **Previous pregnancy loss** |  |  |
| 1 | -0.534 (-0.932, -0.137) | 0.008 |
| 2 | -0.360 (-0.530, -0.191) | < 0.001 |
| ≥ 3 | -0.017 (-0.262, 0.229) | 0.894 |
| **Previous live birth** |  |  |
| 0 | -0.324 (-0.480, -0.169) | < 0.001 |
| 1 | -0.114 (-0.504, 0.277) | 0.569 |
| 2 | 0.221 (-0.147, 0.588) | 0.239 |
| **Education level** |  |  |
| Below high school | -0.219 (-0.574, 0.136) | 0.227 |
| Senior high school | -0.302 (-0.836, 0.232) | 0.268 |
| Above high school | -0.308 (-0.475, -0.140) | < 0.001 |

The GEE adjusted for age, body mass index, the number of previous pregnancy losses, and menstrual regularity.

**Table S3**. Subgroup analyses of omega-3 fatty acids and total cholesterol.

|  | **β (95% CI)** | **P‑value** |
| --- | --- | --- |
| **Age, years** |  |  |
| ≤ 35 | 0.123 (-0.062, 0.308) | 0.194 |
| > 35 | 0.585 (0.082, 1.087) | 0.023 |
| **Body mass index, kg/m^2^** |  |  |
| < 18.5 | 0.067 (-0.140, 0.274) | 0.524 |
| 18.5-24.9 | 0.206 (-0.005, 0.416) | 0.056 |
| ≥ 25 | 0.038 (-0.359, 0.434) | 0.852 |
| **Menstrual regularity** |  |  |
| Yes | 0.109 (-0.074, 0.293) | 0.243 |
| No | 0.420 (-0.188, 1.028) | 0.175 |
| **Previous pregnancy loss** |  |  |
| 1 | 0.227 (-0.163, 0.616) | 0.254 |
| 2 | 0.012 (-0.196, 0.221) | 0.909 |
| ≥ 3 | 0.483 (0.191, 0.775) | 0.001 |
| **Previous live birth** |  |  |
| 0 | 0.138 (-0.043, 0.318) | 0.134 |
| 1 | 0.511 (-0.351, 1.372) | 0.245 |
| 2 | 0.271 (-0.063, 0.606) | 0.112 |
| **Education level** |  |  |
| Below high school | -0.289 (-1.002, 0.423) | 0.426 |
| Senior high school | -0.091 (-0.474, 0.292) | 0.642 |
| Above high school | 0.258 (0.082, 0.435) | 0.004 |

The GEE adjusted for age, body mass index, the number of previous pregnancy losses, and menstrual regularity.

**Table S4**. Subgroup analysis of omega-3 fatty acids and low-density lipoprotein cholesterol.

|  | **β (95% CI)** | **P‑value** |
| --- | --- | --- |
| **Age, years** |  |  |
| ≤ 35 | -0.019 (-0.148, 0.109) | 0.771 |
| > 35 | 0.294 (-0.107, 0.694) | 0.151 |
| **Body mass index, kg/m^2^** |  |  |
| < 18.5 | -0.219 (-0.466, 0.028) | 0.083 |
| 18.5-24.9 | 0.041 (-0.099, 0.181) | 0.565 |
| ≥ 25 | -0.023 (-0.321, 0.274) | 0.878 |
| **Menstrual regularity** |  |  |
| Yes | -0.018 (-0.144, 0.108) | 0.782 |
| No | 0.115 (-0.335, 0.565) | 0.617 |
| **Previous pregnancy loss** |  |  |
| 1 | 0.053 (-0.235, 0.340) | 0.720 |
| 2 | -0.068 (-0.214, 0.078) | 0.359 |
| ≥ 3 | 0.139 (-0.149, 0.427) | 0.345 |
| **Previous live birth** |  |  |
| 0 | -0.014 (-0.141, 0.114) | 0.831 |
| 1 | 0.254 (-0.288, 0.796) | 0.358 |
| 2 | 0.292 (0.092, 0.492) | 0.004 |
| **Education level** |  |  |
| Below high school | -0.289 (-0.685, 0.107) | 0.152 |
| Senior high school | -0.094 (-0.395, 0.208) | 0.542 |
| Above high school | 0.063 (-0.069, 0.194) | 0.350 |

The GEE adjusted for age, body mass index, the number of previous pregnancy losses, and menstrual regularity.

**Table S5**. Subgroup analysis of omega-3 fatty acids and high-density lipoprotein cholesterol.

|  | **β (95% CI)** | **P‑value** |
| --- | --- | --- |
| **Age, years** |  |  |
| ≤ 35 | 0.017 (-0.092, 0.127) | 0.757 |
| > 35 | 0.003 (-0.187, 0.193) | 0.974 |
| **Body mass index, kg/m^2^** |  |  |
| < 18.5 | 0.085 (-0.138, 0.307) | 0.455 |
| 18.5-24.9 | 0.021 (-0.100, 0.142) | 0.736 |
| ≥ 25 | -0.020 (-0.136, 0.096) | 0.735 |
| **Menstrual regularity** |  |  |
| Yes | 0.015 (-0.100, 0.130) | 0.802 |
| No | 0.064 (-0.135, 0.263) | 0.527 |
| **Previous pregnancy loss** |  |  |
| 1 | 0.100 (-0.145, 0.345) | 0.425 |
| 2 | -0.033 (-0.143, 0.077) | 0.555 |
| ≥ 3 | 0.101 (-0.054, 0.257) | 0.202 |
| **Previous live birth** |  |  |
| 0 | 0.029 (-0.081, 0.139) | 0.606 |
| 1 | 0.015 (-0.272, 0.301) | 0.921 |
| 2 | -0.219 (-0.442, 0.003) | 0.054 |
| **Education level** |  |  |
| Below high school | -0.121 (-0.340, 0.098) | 0.280 |
| Senior high school | -0.105 (-0.318, 0.107) | 0.332 |
| Above high school | 0.055 (-0.065, 0.176) | 0.366 |

The GEE adjusted for age, body mass index, the number of previous pregnancy losses, and menstrual regularity.
